# Single level posterolateral lumbar fusion in a New Zealand White rabbit (*Oryctolagus cuniculus*) model: Surgical anatomy, operative technique, autograft fusion rates, and perioperative care

**DOI:** 10.1002/jsp2.1135

**Published:** 2020-12-23

**Authors:** James D. Crowley, Rema A. Oliver, Michael J. Dan, Daniel J. Wills, John W. Rawlinson, Rebekah A. Crasto, James M. O'Connor, Gregory J. Mitchell, Christopher J. Tan, William R. Walsh

**Affiliations:** ^1^ Surgical and Orthopaedic Research Laboratories, Prince of Wales Clinical School University of New South Wales Sydney, Prince of Wales Hospital Sydney New South Wales Australia; ^2^ Sydney Veterinary Emergency and Specialists Sydney New South Wales Australia

**Keywords:** autograft, bone graft, fusion, intertransverse, posterolateral, rabbit, spine, vertebrae

## Abstract

**Introduction:**

The posterolateral lumbar fusion (PLF) New Zealand White (NZW) (*Oryctolagus cuniculus*) rabbit model is a long‐standing surgical technique for the preclinical evaluation of materials for spinal fusion. A detailed understanding of lumbar spine anatomy and perioperative care requirements of rabbits is imperative for correct execution of the model both scientifically and ethically. This study describes the preoperative procedures and surgical techniques used in single level PLF in a NZW rabbit model as it pertains to the animal husbandry, lumbar spine anatomy, anesthesia, surgical approach, and perioperative care of rabbits in a research setting.

**Materials and Methods:**

We describe the surgical technique and perioperative patient care for single level PLF in a NZW rabbit model. Medical records from a single research facility were retrospectively reviewed for adult NZW rabbits that underwent single level PLF (L4‐L5) between January 2016 and December 2019. The number of lumbar vertebrae per rabbit, fusion rates at 12 weeks using iliac crest autograft and complications are reported. Skeletal maturity was confirmed by preoperative fluoroscopic and radiographic documented closure of hindlimb physes.

**Results:**

The PLF rabbit surgical model and perioperative patient care is described. PLF was performed in 868 adult female entire NZW rabbits. The majority of rabbits had seven lumbar vertebrae (620/868; 71.4%), followed by six (221/868; 25.5%), and eight (27/868; 3.1%). Fusion rates at 12 weeks for PLF using iliac crest autograft as assessed by manual palpation and radiographic assessment was 76.9% and 70.0%, respectively. Postoperative complications included occasional partial autograft site wound dehiscence due to self‐trauma.

**Conclusions:**

For PLF rabbit models, a detailed understanding of the surgical technique, rabbit lumbar anatomy including number of lumbar vertebrae, and dietary and husbandry requirements of rabbits, is essential for execution of the model and animal welfare.

## Graphical abstract

Descriptive report of the surgical technique and perioperative patient care for single level posterolateral lumbar fusion (PLF) in a New Zealand White rabbit model, and a retrospective account of variation in number of lumbar vertebrae in rabbits. The majority of rabbits had seven lumbar vertebrae (620/868; 71.4%), followed by six (221/868; 25.5%), and eight (27/868; 3.1%). Mean spinal fusion rates at 12 weeks for bilateral iliac crest autograft alone were 80/104 (76.9%) and 73/104 (70.0%) for manual palpation and radiographic assessment (Lenke Grade A), respectively. For PLF rabbit models, a detailed understanding of the surgical technique, rabbit lumbar anatomy including number of lumbar vertebrae, and dietary and husbandry requirements of rabbits, is essential for animal welfare and experimental procedures.

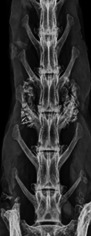

## 1 INTRODUCTION

The New Zealand White (NZW) (*Oryctolagus cuniculus*) rabbit model of posterolateral lumbar fusion (PLF) has been widely used for spinal fusion research since it was first described by Boden et al.^1^ The gold standard for successful spinal fusion is traditional autogenous iliac bone graft, which has a reported fusion rate ranging from 50% to 90%.^2^, ^3^, ^4^, ^5^, ^6^ The NZW rabbit PLF model is not stabilized with any posterior fixation and is considered a noninstrumented model that has been used for evaluation of the in vivo performance of bone graft substitutes and biological materials,^2^, ^3^, ^4^, ^5^, ^7^ as well as the effect of medications such as opioids,^8^ nonsteroidal anti‐inflammatories,^9^ and antibiotics^10^ on spinal fusion. A complete list of the studies using this model is beyond the scope of this manuscript.

Regardless of the purpose of the study, the investigator(s) and surgeon(s) must have a comprehensive understanding of the surgical technique, the anesthetic protocol, and pre and postoperative care requirements of rabbits, specifically diet and husbandry. Failure to address these aspects of the study may lead to complications and associated morbidity and/or mortality which results in increased costs and time requirements of the experiment.^11^ Common complications of PLF include incorrect level of fusion, neurological impairment, hemorrhage, surgical site infection and anesthesia‐related issues.^11^, ^12^

Palumbo et al have described detailed surgical anatomy for PLF in the rabbit.^11^ Valdes et al reported a 26% complication rate for PLF in a cohort of 48 rabbits.^13^ The authors adapted this knowledge to describe a refined experimental protocol without complication. The authors attributed the reduction in complication rate to a more detailed understanding of the anatomy and improvements in anesthesia and postoperative care procedures. Despite these studies, based on our extensive experience in PLF, there remains a need to document additional measures to ensure accuracy of surgical technique and describe the unique dietary and husbandry requirements of the rabbit with the aim of improving animal welfare. Therefore, the purpose of this study was to share our experimental protocol of PLF in a large rabbit cohort, with a focus on lumbar vertebral body anatomy, methods for identification of arthrodesis level, anesthesia and pre and postoperative care to provide researchers with a detailed description of this model in our hands.

## 2 MATERIALS AND METHODS

Medical records of adult female entire NZW rabbits that underwent single level PLF (L4‐L5) between January 2016 and December 2019 were reviewed. The number of lumbar vertebrae per rabbit as determined by fluoroscopic and radiographic assessment was recorded.

### 2.1 Experimental protocol

Our experimental protocol was implemented in accordance with the ASTM Standard Guide for in vivo evaluation of rabbit lumbar intertransverse process spinal fusion models^14^ following institutional ethics approval (Animal Ethics Committee, UNSW, Sydney).

### 2.2 Preoperative assessment

#### 2.2.1 Physical examination

Prior to study enrolment, all rabbits were physically examined by a veterinarian familiar with rabbit husbandry, anatomy, physiology, and surgery. Routine parameters were assessed including mucous membrane color, capillary refill time, heart rate, respiratory rate, presence of gastrointestinal sounds, and rectal temperature. Close attention was paid to mentation, body condition, coat condition and ambulation. All rabbits experienced a minimum 1‐week acclimatization period prior to surgical intervention to allow for adjustment to their environment, including pen mates and attending staff.

#### 2.2.2 Skeletal maturity

Skeletal maturity was confirmed prior to enrolling an animal for PLF by fluoroscopic and radiographic documentation of distal femoral, proximal tibial (epiphyseal and apophyseal), and proximal fibular physeal closure. A portable fluoroscopy unit (Shanghai Bojin Electric Instrument & Device Co, Shanghai, China) was used for physeal closure screening at the time of physical examination. Immediately prior to surgery, mediolateral radiographs were taken to further document physeal closure. Radiographs were taken using a POSKOM model PXP‐60HF portable machine. Digital cassettes (AGFA CR MD4.0 Cassette, AGFA, Germany) were processed by an AGFA CR 75.0 Digitizer and workstation.

#### 2.2.3 Anesthesia and perioperative care

All rabbits were weighed prior to surgery to ensure correct calculation of all medications to be administered. The rabbits were premedicated with Buprenorphine (0.03 mg/kg) and Midazolam (0.5 mg/kg) via intramuscular injection using a 23 g needle. The rabbits were preoxygenated for 10 minutes prior to masked isoflurane induction and maintained between 2% and 3%, titrated to effect. A balanced crystalloid solution (Hartmann's solution) was administered subcutaneously (20 mL/kg) prior to commencement of surgery. Continuous multiparameter cardiopulmonary monitoring (Datalys V7, Lutech, Ronkonkoma, New York) was used throughout anesthesia and recorded every 10 minutes during the procedure. The isoflurane setting was weaned nearing the completion of the procedure. The rabbits received external heat support in the form of a heat pad. Following completion of surgery, the rabbits were wrapped in a clean towel for added warmth and received masked oxygen supplementation until righting before being returned to their housing pen. All rabbits received perioperative prophylactic antibiotics (Enrofloxacin 5 mg/kg IM or Procaine Penicillin 50 000 IU/KG IM for 5 days duration), perioperative and intraoperative opioid analgesia (Buprenorphine 0.03 mg/kg IM at induction then PRN), and postoperative nonsteroidal anti‐inflammatory medication (Meloxicam 0.85 mg/kg q 12‐24 hours for 5 days).^15^ Rabbits were examined daily for the first 7 days postoperative by trained staff familiar with the animals prior to surgery with additional analgesia provided as required. Rabbits were observed daily and weighed once weekly for the remainder of the relevant study period(s). The anesthetic, perioperative and acute monitoring forms used at our institution are provided in Appendix 1 and 2.

#### 2.2.4 Fluoroscopic lumbar vertebrae assessment

**FIGURE 1:**
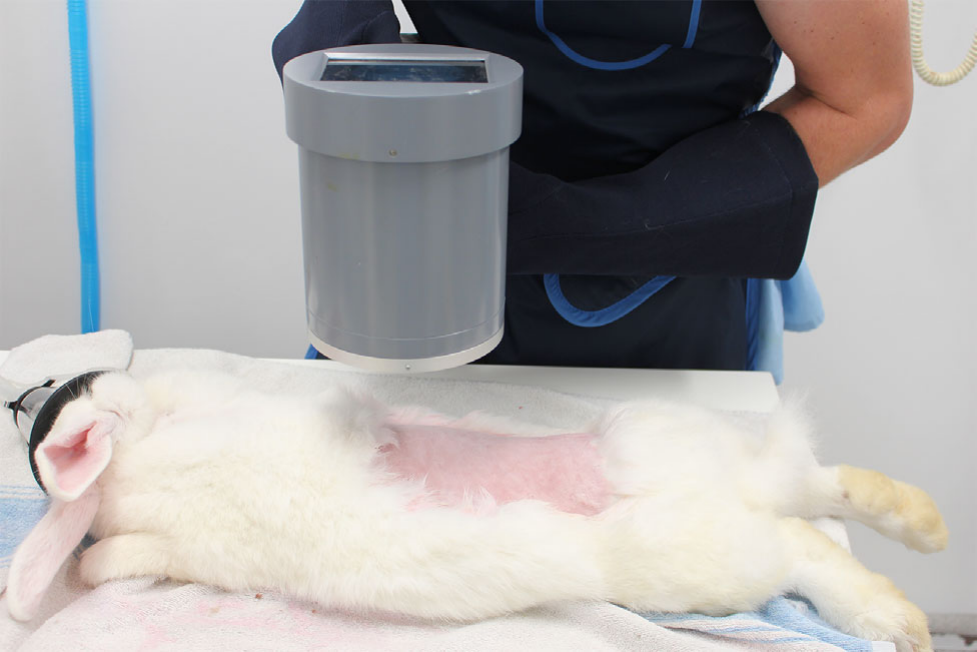
Preoperative fluoroscopic assessment for determination of number of lumbar vertebrae and vertebral anomaly screening

Prior to surgery, a portable fluoroscopy unit (Shanghai Bojin Electric Instrument & Device Co, Shanghai, China) was used to count the number of lumbar vertebrae and screen for any structural anomalies such as hypoplastic vertebral bodies (Figure 1).

### 2.3 Surgery

#### 2.3.1 Surgical site preparation (PLF and iliac crest corticocancellous bone graft harvest)

The surgical fields for PLF and iliac crest corticocancellous bone graft harvest were prepared under aseptic conditions. A wide area of fur was clipped from the dorsum of the rabbit, extending caudally from the last rib to the tail base and laterally to a level approximately a quarter of the way down the flank, ventral to the wing of the ilium. The area was aseptically prepared with chlorhexidine‐alcohol soaked gauze swabs followed by povidine‐iodine spray. The surgical field was then draped in a sterile fashion.

#### 2.3.2 Bone graft harvest (iliac crest)

**FIGURE 2:**
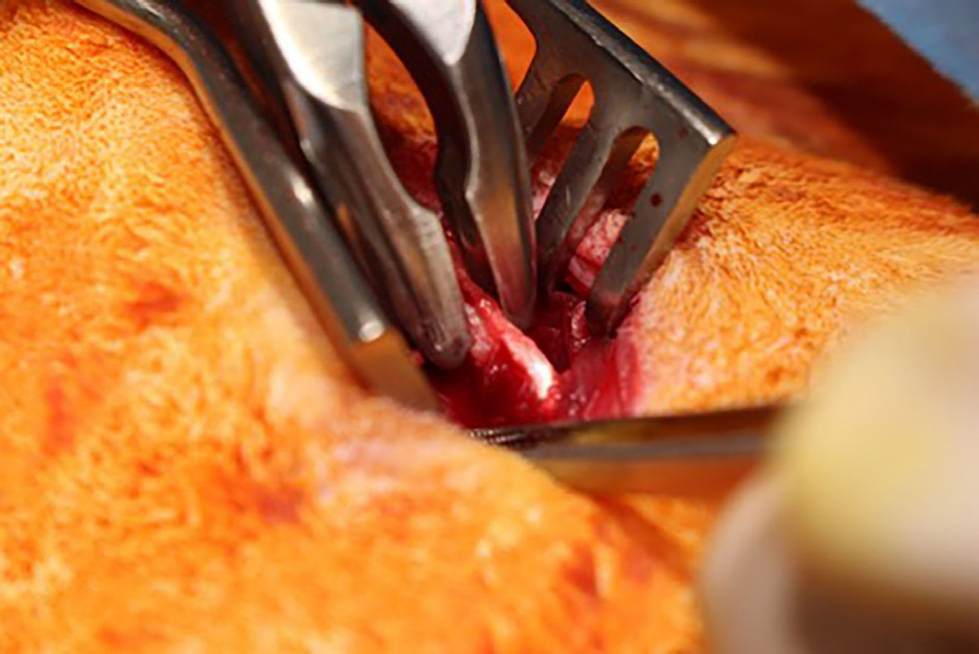
Corticocancellous bone graft harvest from the iliac wing using bone rongeurs

**FIGURE 3:**
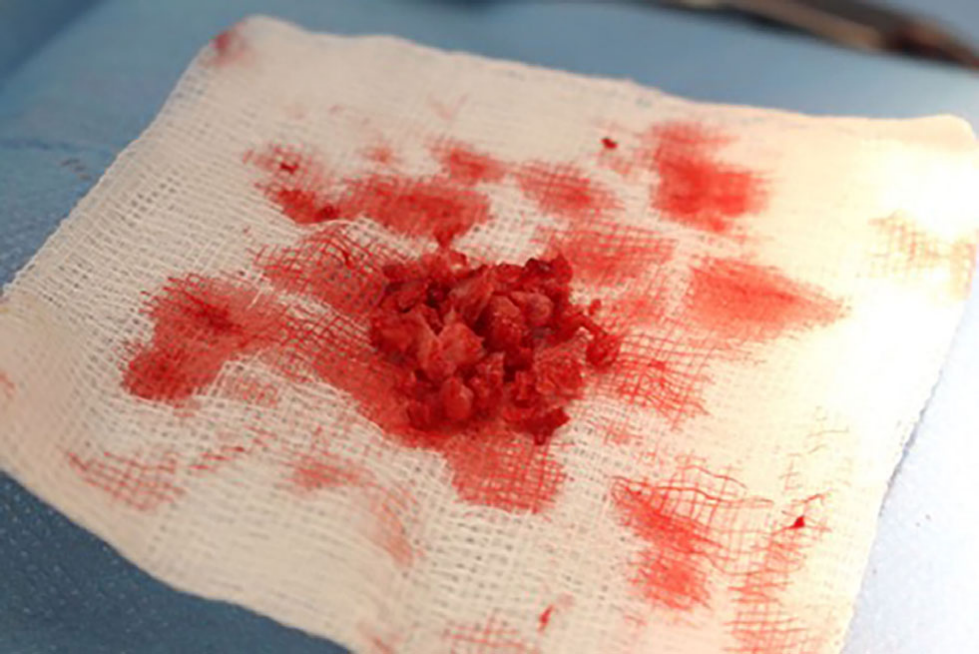
Two cc (~1.7 g) of corticocancellous bone graft harvested from the iliac crest

A linear skin incision was made along the spine of the iliac crest (left or right) in a caudomedial to craniolateral direction. Subcutaneous and fascial tissue was incised using a combination of sharp and blunt dissection. The gluteal muscles were incised along their fascial origin and elevated from the cranial and caudal aspects of the iliac crest, taking care to stay as close to the ilium as possible to minimize tissue trauma and associated hemorrhage. The authors find it useful to place a gauze swab on either side of the iliac crest using a Freer periosteal elevator^16^ to assist in hemostasis and removal of muscle and connective tissue prior to harvesting of the corticocancellous graft. Graft harvest should be confined to the cranial two thirds of the iliac crest as manipulation of the caudal third, close to the sacroiliac junction, may result in damage to the lumbosacral trunk and cranial gluteal neurovasculature. Bone graft was harvested using bone rongeurs (Figure 2). Care was taken to ensure that the corticocancellous bone harvested was free of soft tissue as well as excessive blood which can be a source of variation when quantifying the amount. Sharp dissection was used to remove redundant soft tissue and the graft was morselized into small pieces (<5 mm)^3^ (Figure 3). For cases where an alternative graft material was being evaluated, 2 cc (which equates to approximately 1.7 g) of autograft was harvested unilaterally. This quantity is used to ensure a reproducible measurement of autograft as particle shape and packing can influence volume measurements. Autograft was then combined evenly with 2 cc of material as required. For positive control (autograft only) cases, 2 cc was harvested bilaterally. Muscle at the harvest site was closed using 3‐0 monofilament suture material in a simple continuous pattern. The subcutaneous layers were closed in simple continuous buried pattern with 4‐0 monofilament absorbable material. The skin was closed with 4‐0 monofilament absorbable material in either a buried simple continuous or Ford‐interlocking pattern.

#### 2.3.3 Bone marrow aspiration (proximal tibia)

**FIGURE 4:**
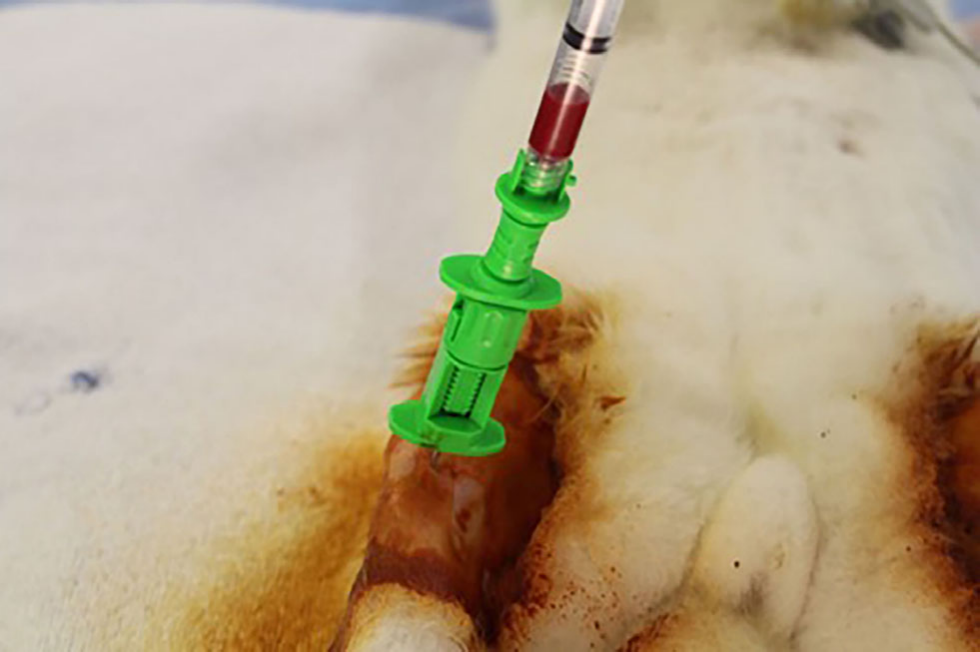
Bone marrow aspiration from the proximal tibia using a 25 mm, 18 g trochar needle (Sternobell, Biopsybell, Mirandola, Italy))

Bone marrow aspirate (BMA), when required for the study, was harvested from the proximal tibia for hydration of select bone graft substitutes prior to implantation. A 25 mm, 18 g trochar needle (Sternobell, Biopsybell, Mirandola, Italy) was manually inserted into the cancellous bone of the proximo‐medial tibia (Figure 4). The insertion site was approximately at the midpoint of the tibial tuberosity cranially and the caudal cortex of the tibia caudally. A release in pressure can be felt once the trochar penetrates the near cortex and reaches the bone marrow cavity. The stylet was removed, a sterile Luer‐lock 3 mL syringe attached to the hub of the trochar and negative pressure applied to harvest 2 mL of bone marrow. The trochar was removed and firm manual pressure placed over the site for hemostasis. BMA hydration of the materials for a study was performed immediately following harvest to avoid the use of any antithrombotic agents.

#### 2.3.4 PLF surgical approach

Following appropriate draping of the surgical field, the surgeon(s) determined the L4‐L5 level for fusion via counting of the dorsal spinous processes using manual palpation based on the number of vertebrae found during the preoperative preparation. The surgeon(s) identified the lumbosacral junction, then moved cranially to L4‐L5. Intraoperative fluoroscopy can be used to clarify the L4‐L5 level if required.

**FIGURE 5:**
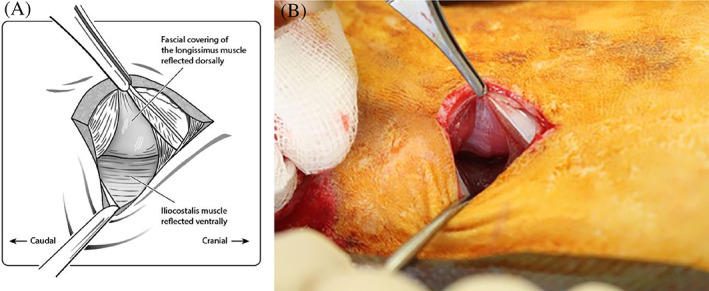
Schematic illustration (A) and intraoperative appearance (B) of the intermuscular plane between the longissimus and iliocostalis muscles

A dorsal midline skin incision, approximately 4 to 5 cm in length, was made centered over the L4‐L5 level. Subcutaneous tissue and fat were undermined using blunt dissection. Approximately 2 cm lateral to the midline, a white line can be visualized in the longissimus muscle. Approximately 2 to 3 mm lateral to this, a 5 cm incision was made deep through the longissimus muscle with a no. 15 scalpel blade. The longissimus muscle was then separated from the iliocostalis muscle by identifying the fascial plane between the two muscle bellies using a Freer periosteal elevator (Figure 5).

**FIGURE 6:**
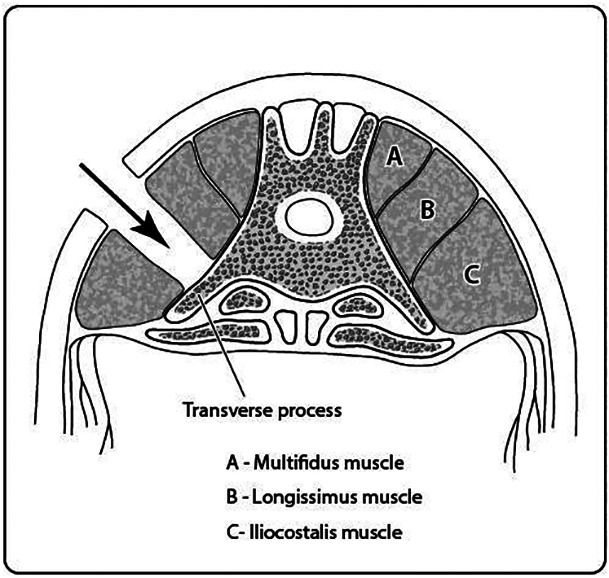
Schematic illustration of the surgical approach to the transverse processes made through the intermuscular plane between the longissimus and iliocostalis muscles. Illustration adapted from Zunariah et al^19^

**FIGURE 7:**
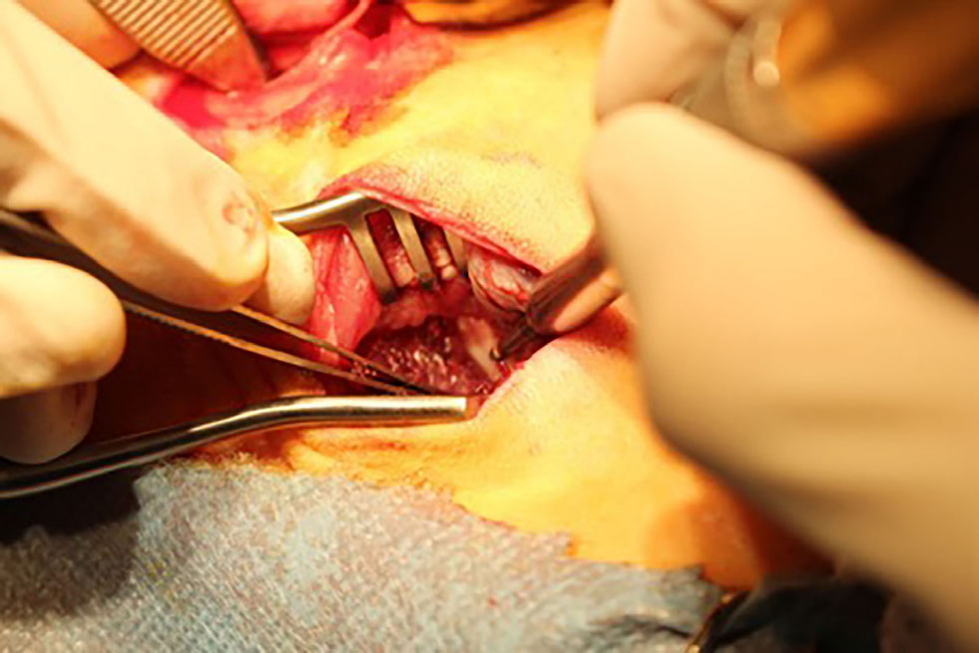
Decortication of L4 and L5 transverse processes using a high‐speed pneumatic surgical drill (Midas Rex, Medtronic, Memphis, TN) with an M‐8 Matchstick Burr

**FIGURE 8:**
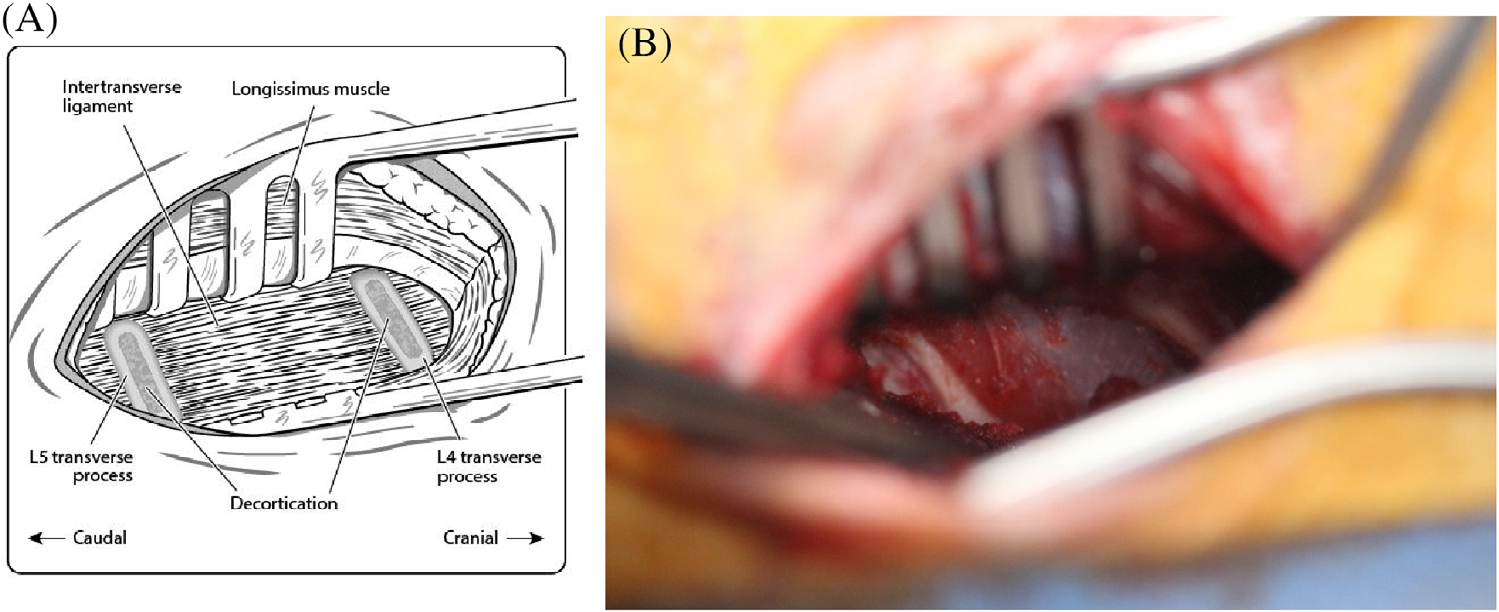
Schematic illustration (A) and intraoperative appearance (B) of the L4 and L5 transverse processes following decortication

A muscular window was then created by carefully placing Weitlaner self‐retaining retractors between the two muscle bellies, elevating the longissimus muscle dorsomedially and the iliocostalis muscle laterally to access the middle third of the transverse processes (Figure 6). It can be useful to pack the surgical site with a gauze swab for hemostasis and removal of additional soft tissue overlying the transverse processes. Using a Freer periosteal elevator, the transverse processes and intertransverse ligament were exposed by manually elevating remaining muscle fibers, taking care to avoid the segmental artery which courses laterally from the cranial aspect of the transverse process. Decortication was performed using a high‐speed pneumatic surgical drill (Midas Rex, Medtronic, Memphis, Tennessee*)* with an M‐8 Matchstick Burr. The central portion of the transverse processes were carefully decorticated for a distance of 10 mm from the vertebral body and pars to a level where bleeding bone beds were visually present to the surgeon^3^ (Figures 7 and 8). Great care was taken to decorticate the bone, creating a bleeding bone bed in preparation for placement of graft material over the transverse processes. Given the oblique cranioventral angulation of the transverse processes, ambidexterity is advantageous for decortication. The same procedure was performed on the contralateral side. To facilitate accurate bilateral approach, the location of the L4 and L5 transverse processes were marked with sterile needles through the longissimus muscle to ensure the correct level was treated on both sides.

#### 2.3.5 Material placement

**FIGURE 9:**
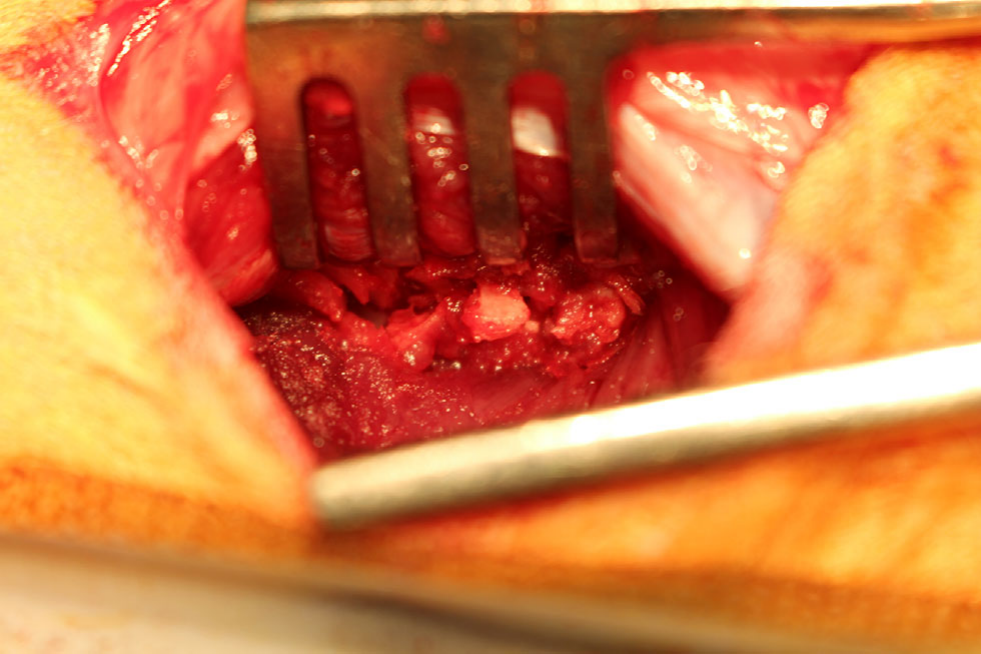
Material placement on the decorticated transverse processes and overlying the intertransverse ligament between the L4 and L5 transverse processes

Material (bone graft +/− synthetic material of 2 cc per side) for implantation was placed into 3 cc syringes with the tip removed to provide a simple means to accurately place the materials in the prepared posterolateral beds. The material was implanted bilaterally on the decorticated transverse processes and overlying the intertransverse ligament between the L4 and L5 transverse processes (Figure 9). Care was taken to ensure the material was placed midline and directly on the decorticated transverse processes.

#### 2.3.6 Wound closure

The muscle planes were closed using 3‐0 monofilament absorbable suture in a simple continuous pattern. Care was taken to take deep bites of the muscle to ensure closing of dead space to prevent graft migration and seroma formation. The superficial fascia of the longissimus and multifidus muscles was included in this layer to assist in closing the dead space. The subcutaneous layer was closed in a simple continuous buried pattern with 4‐0 monofilament absorbable material. The skin was closed with 4‐0 monofilament absorbable material in either a buried simple continuous or Ford‐interlocking pattern. A transparent adherent film spray (Op‐site, Smith & Nephew) was applied to all surgical wounds to encourage formation of a fibrin seal and discourage self‐trauma.

#### 2.3.7 Postoperative radiography

Dorsoventral lumbar digital radiographs were performed at the completion of surgery to confirm appropriate material placement as well as provide immediate postoperative radiographic data on the radio‐opacity of the treated level for future comparisons. Radiographs were taken using a POSKOM model PXP‐60HF portable machine. Digital cassettes (AGFA CR MD 4.0 Cassette, AGFA, Germany) were processed by an AGFA CR 75.0 Digitizer and workstation.

### 2.4 Spinal fusion assessment

#### 2.4.1 Manual palpation

Immediately following harvest, the stability of the lumbar spine was assessed by manual palpation by two trained and experienced observers in a blinded manner according to Professor Boden et al.^1^ Briefly, the treated motion segment was assessed in lateral bending and flexion/extension and compared to the cranial and caudal motion segments. The motion segments at L4‐L5 were graded as either fused (rigid, no detectable movement at the intervertebral disc space) or not fused (not rigid, movement detected at the intervertebral disc space).

#### 2.4.2 Radiographic assessment

Following harvest, the spines were immediately radiographed in the dorsoventral plane using a Faxitron (Faxitron Bioptics LLC, Arizona) and digital cassettes (described previously). Radiographs were processed by a dedicated workstation (described previously). The DICOM data was converted to bitmap images using DICOM Works (ezDICOM medical viewer, 2002). Radiographs were reviewed by blinded observers and fusion was graded according to the Lenke scale.^17^ Bilateral fusion was defined as a Grade A, where it was deemed definitely solid with bilateral robust bridging bone. The Lenke grading scale was originally described for autograft fusion for human patients with scoliosis.^17^

Fusion was also assessed by micro computed tomography and biomechanical testing (range of motion) on the day of euthanasia, and subsequently processed for histology and histomorphometry. The samples are never frozen. A discussion of these experimental endpoints is beyond the scope of this manuscript.

### 2.5 Diet and husbandry

All dietary and husbandry protocols were implemented in accordance with the Animal Research Review Panel (ARRP) guidelines for the housing of rabbits in scientific institutions.^18^ Rabbits were housed in groups of 3‐4 within 3‐3.2 m^2^ enclosed pens, floored with hay to a depth of at least 5 cm. A wooden hutch shelter was provided for each rabbit. Cardboard boxes, toilet rolls and plastic balls were provided for environmental enrichment. Close attention was paid to conflict between rabbits, with offending rabbits singularly housed with clear vision of other rabbits or rotated between pens as required.

All rabbits were fed a high‐quality diet, of which the majority was high‐fiber timothy or oaten hay, supplemented with leafy greens such as silver beet, spinach, bok choy, pak choy, and broccoli. High‐quality commercial pellets, herbs (dill, parsley, coriander, mint, etc) and fruits (strawberries, apple, etc) were provided in moderation. Water was provided ad libitum via self‐automated bottles. Calm, slow‐paced music was played during daylight hours.

## 3 RESULTS

### 3.1 Number of lumbar vertebrae

**FIGURE 10:**
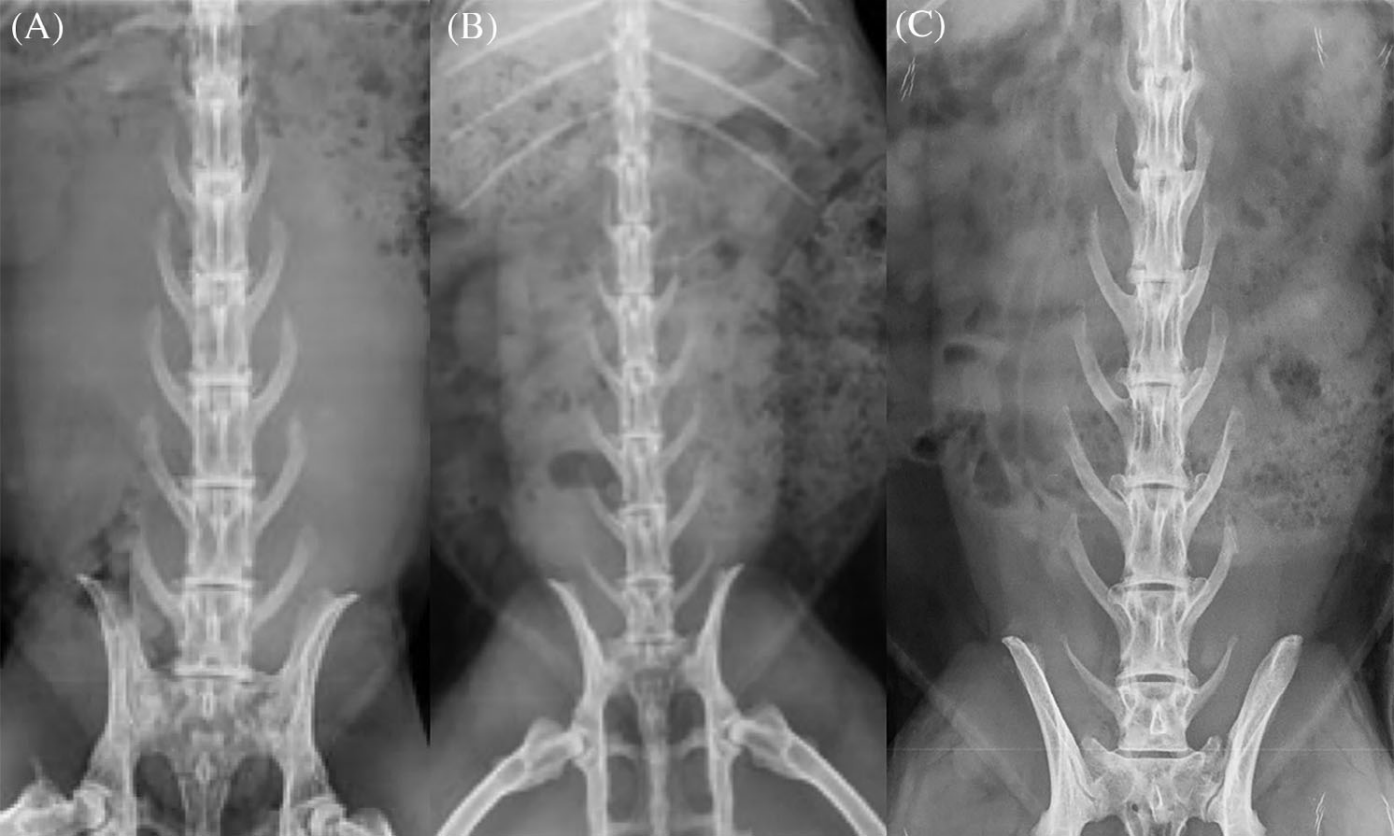
Dorsoventral radiographs demonstrating anatomical variation in total number of lumbar vertebrae in New Zealand White rabbits that underwent single level L4‐L5 posterolateral lumbar fusion; (A) six, (B) seven, (C) eight

PLF was performed in 868 adult female entire NZW rabbits across 30 preclinical studies. The most common number of lumbar vertebrae was seven (620/868; 71.4%), followed by six (221/868; 25.5%), and eight (27/868; 3.1%) (Figure 10, Table 1).

**TABLE 1:** Distribution of number of lumbar vertebrae in 868 NZW rabbits that underwent single level L4‐L5 PLF between January 2016 and December 2019

Lumbar vertebrae	Total	Percentage (%)
Six	221	25.5
Seven	620	71.4
Eight	27	3.1
	868	100

Abbreviations: NZW, New Zealand White; PLF, posterolateral lumbar fusion.

### 3.2 PLF using bilateral iliac crest bone graft

Bilateral autograft fusion with a 12 week timepoint was performed in 104/868 (11.9%) rabbits across 13/30 preclinical studies. The remaining 764 rabbits underwent L4‐L5 PLF with autograft only at an earlier timepoint or with various combinations of autograft and synthetic material or synthetic material only, as dictated by the study design. Typical sample size was n = 8 per group per timepoint.

### 3.3 Radiographic analysis

**FIGURE 11:**
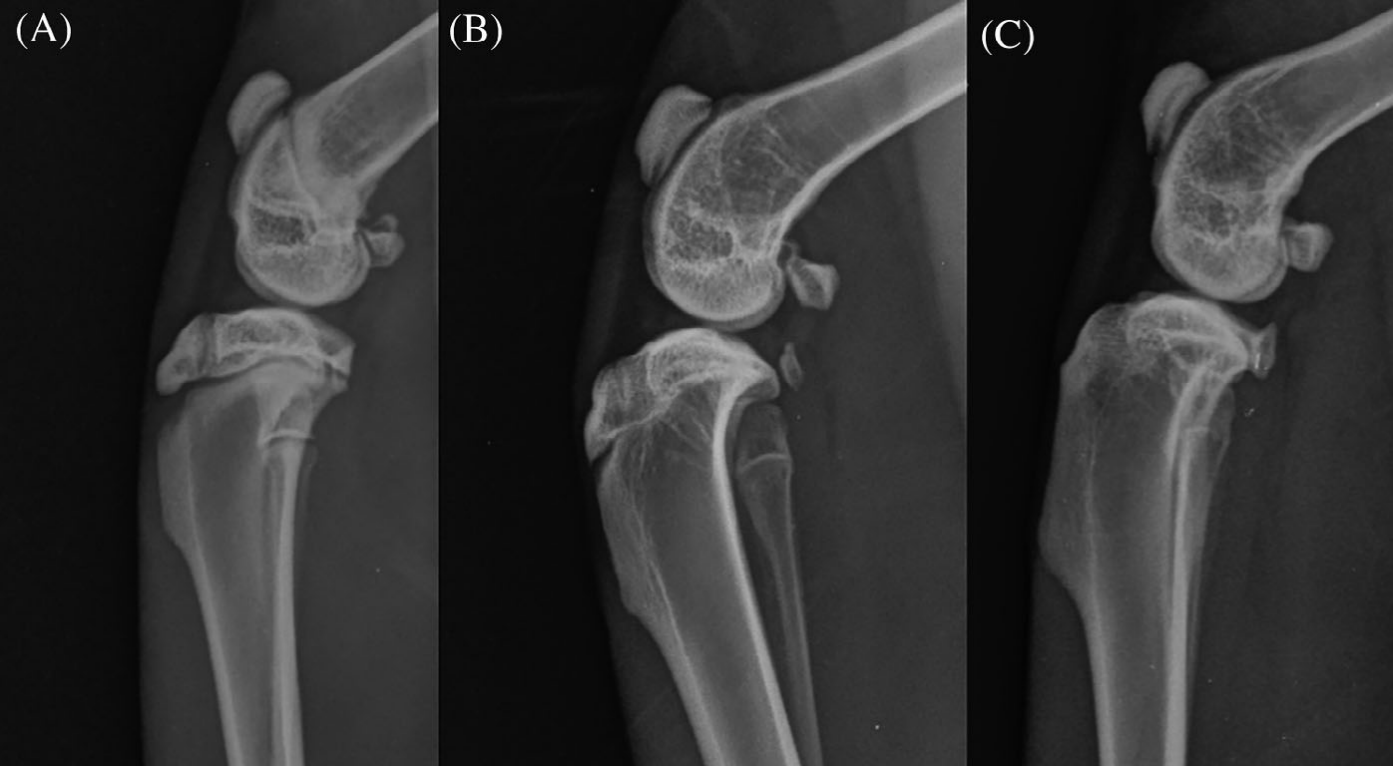
Mediolateral stifle radiographs demonstrating distal femoral, proximal tibial (apophyseal and epiphyseal) and proximal fibular physes status of New Zealand White rabbits at 3, 6, and 12 months of age. (A) 3‐months‐old: note all physes are open. (B) 6‐months‐old: closed distal femoral, proximal tibial epiphyseal and proximal fibular physes. The tibial apophyseal physis is open. (C) 12‐months‐old. All physes are closed

**FIGURE 12:**
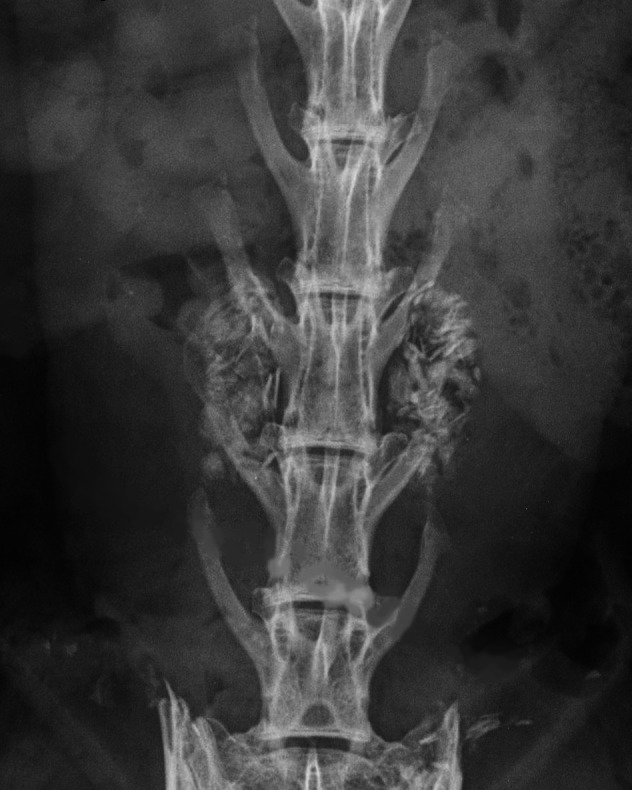
Immediate postoperative dorsoventral radiograph following L4‐L5 posterolateral lumbar fusion with bilateral iliac crest autograft harvest in a New Zealand White rabbit with 6 lumbar vertebrae. Note: symmetrical bilateral autograft placement close to midline

All animals were confirmed to be skeletally mature prior to surgery as verified by closure of the distal femoral, proximal tibial and fibular physes. Representative radiographs are presented in Figure 11. Accurate implant placement at the L4‐L5 level was confirmed in all cases based on postoperative radiographs (Figure 12).

### 3.4 Fusion assessment for autograft PLF at 12 weeks

**FIGURE 13:**
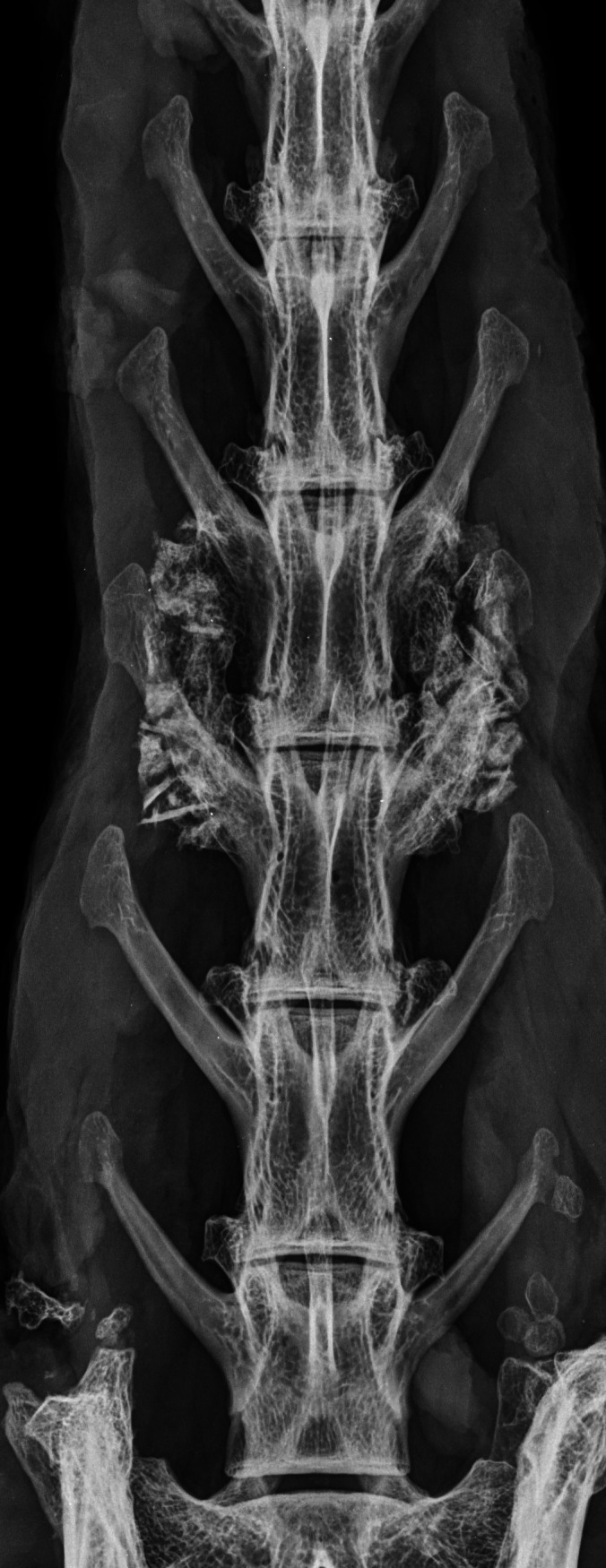
Dorsoventral Faxitron radiograph of a fused 12 week L4‐L5 posterolateral lumbar fusion with bilateral iliac crest autograft harvest in a New Zealand White rabbit with 7 lumbar vertebrae. Note: complete bilateral bridging bone spanning the L4 and L5 transverse processes (Lenke Grade A)

Mean spinal fusion rates at 12 weeks for bilateral iliac crest autograft alone were 80/104 (76.9%) and 73/104 (70.0%) for manual palpation and radiographic assessment (Lenke Grade A), respectively. A Faxitron radiograph of a fused L4‐L5 PLF with autograft at 12 weeks is provided in Figure 13.

### 3.5 Surgical time

In our experience with this preclinical model, PLF with unilateral autograft harvest in combination with synthetic material is an approximately 30 to 40 minutes procedure, that is comprised of unilateral autograft harvest (5‐10 minutes), PLF (20 minutes) and wound closure (5‐10 minutes). PLF with bilateral autograft harvest is an approximately 50 to 60 minute procedure, including bilateral autograft harvest (10‐20 minutes), PLF (20 minutes) and wound closure (10‐15 minutes). Total surgical time is dependent on the required preparation of the synthetic graft material (hydration, mixing, etc). Reporting of surgical times for each synthetic material is beyond the scope of this manuscript.

### 3.6 Complications

All rabbits maintained or gained weight during their experimental lifetime. There were no anesthetic‐related complications. We observed occasional episodes of partial dehiscence of the autograft harvest site due to self‐trauma, that were managed conservatively with Elizabethan collars. We did not observe neuropraxia or other neurological complications. No asymmetric material implantation, fracture of transverse processes, or surgical site infections were experienced.

## 4 DISCUSSION

We describe the preoperative care and procedures, variation in lumbar vertebrae, skeletal maturity assessment, surgical approach, autograft fusion rates and perioperative care of the single level PLF in the preclinical NZW rabbit model. Originally described by Professor Boden et al,^1^ the NZW PLF rabbit model of spinal fusion has been extensively used by researchers around the world to study the biology of spinal fusion and the in vivo response of bone graft materials. While the model does not have any posterior fixation, it is widely accepted with comparable posterolateral fusion rates to humans using autograft alone. Animal welfare must be the primary concern when designing and executing all preclinical experimentation. Regardless of the experimental purpose, an intricate knowledge of the PLF procedure, surgical anatomy, and anesthetic and husbandry requirements of rabbits, is required to ensure accurate surgical execution, minimize perioperative complications and their associated time and financial burdens. Considering the complex nature of this model, a team approach with a wide skill set and training is critical to achieve a positive outcome, including animal housing facility and husbandry staff, animal technicians, surgeons and personnel responsible for postoperative monitoring and management.

The overall mean fusion rates based on manual palpation and radiographic assessment (Lenke Grade A) for PLF with bilateral iliac crest autograft at a 12 week timepoint were 76.9% and 70.0%, respectively. Our results compare favorably with previous reports using autograft alone in PLF.^2^, ^3^, ^4^, ^5^, ^6^ Riordan et al (2013) reported a meta‐analysis of autograft spinal fusions in 733 rabbits across 56 PLF studies.^6^ The authors reported an overall autograft L4‐L5 fusion rate of 53.9% from 97 rabbits for timepoints ranging from 1 to 26 weeks. The authors concluded that fusion occurs reliably in 5 weeks, based on 64.6% fusion rate in 96 rabbits at 5 weeks and 56.4% in 477 rabbits at timepoints >6 weeks; however, no further breakdown of these timepoints was provided. In our experience, a 12 week timepoint is a robust time period to assess fusion, allowing for bone remodeling and spanning of the transverse processes. Earlier timepoints are insufficient to achieve reliable fusion and should be interpreted with caution.

A common complication of PLF is incorrect implantation of the graft material which was not experienced in this cohort. Single level spinal fusion is commonly performed bilaterally at the L3‐L4, L4‐L5, or L5‐L6 levels in preclinical rabbit models.^1^, ^4^, ^5^, ^6^, ^11^, ^13^, ^19^ Symmetrical bilateral placement of material is vital to the outcome of the experiment. In our experience, knowledge of lumbar anatomy and preoperative fluoroscopic assessment is vital to ensure correct placement. The number of lumbar vertebrae in rabbits is variable and should be determined prior to surgery. Of the 868 rabbits that underwent spinal fusion at our facility, the majority of rabbits had seven lumbar vertebrae (71.4%), while 25.5% had six and 3.1% had eight. Knowledge of each individual rabbit's number of lumbar vertebrae is critical for correct identification of the L4‐L5 implantation site at the time of surgery. Preoperative fluoroscopy allows for rapid identification of the number of lumbar vertebrae and any anomalies such as malformed or hypoplastic vertebrae. The fluoroscopy unit is portable, allowing for intraoperative clarification of the L4‐L5 level and symmetrical bilateral material implantation. Fluoroscopy, manual vertebrae counting and marking of the correct level with sterile needles provides a simple means to avoid this error.

Appropriate decortication of the transverse processes is important to encourage spinal fusion.^20^ The Midas Rex M‐8 Matchstick burr has cutting lips on the side of the burr, requiring the burr to be held at an oblique angle to the bone to facilitate decortication. A gentle fanning motion of the hand is used to ensure consistent decortication along the central portion of the transverse process. Surgical training prior to the use of the Midas Rex in the rabbit PLF model is a standard part of our facility's procedures to avoid iatrogenic injury and incorrect use.

The traditional rabbit PLF spinal fusion model utilizes the Wiltse approach that is, the intermuscular plane between the multifidus and longissimus muscles, which facilitates direct access to the pars, transverse processes, and facet joints of the lumbar spine.^21^ The surgical approach we describe is via the intermuscular plane between the longissimus and iliocostalis muscles; lateral and ventral relative to the Wiltse approach, as described by Zunariah et al.^19^ We have found that this approach is less traumatic to the soft tissues and results in minimal to no hemorrhage, namely aided by identification and dorsal reflection of the fascial covering of the longissimus muscle. The central portion of the transverse processes are visualized by this approach, with the pars and lateral extremities of the transverse processes not accessed. This restricted intermuscular window avoids unnecessary exposure of the paraspinal bed to prevent graft migration and dead space formation providing a reproducible corridor for implantation.

A detailed understanding of surgical anatomy coupled with a minimally traumatic surgical technique is imperative for correct execution of the procedure to minimize intra and postoperative complications such as hemorrhage, nerve palsy, and surgical site infection. During autograft harvest, hemorrhage can occur when the caudal third of the iliac wing is harvested due to compromise to the lumbosacral trunk and cranial gluteal neurovasculature. We avoid this region and focus our harvest on the lateral aspect of the iliac crest. For the PLF approach, respect for and identification of the fascial plane between the longissimus and iliocostalis muscles minimizes hemorrhage and the associated pain of muscle splitting. Hemorrhage may also result from damage to the segmental spinal vessels as they originate cranial to the transverse processes. Great care is taken during manipulation of the implantation site and decortication of the transverse processes to avoid disrupting these vessels. If hemorrhage is encountered in either of these regions, hemostasis is achieved with gauze swab packing or vessel ligation. Attention is paid to closing dead space at both the autograft harvest and implantation sites by the apposition of tissue planes to minimize the risk of seroma formation which may foster surgical site infection.

Confirming skeletal maturity prior to enrolling an animal in a study is important for study integrity.^14^ The literature remains inconsistent regarding skeletal maturity in rabbits and methods to document closure of hindlimb physes.^22^, ^23^, ^24^, ^25^ Variations in breeder stock and genetics make age and body weight imprecise parameters for determining skeletal maturity in NZW rabbits. Skeletal maturity must be confirmed by preoperative fluoroscopic or radiographic assessment to avoid enrolment of immature animals which could artificially skew the results of fusion given the potential increased healing capacity of younger animals. Figure 11 demonstrates the radiographic appearance of distal femoral, proximal tibial (apophyseal and epiphyseal) and proximal fibular physes of NZW rabbits at 3, 6, and 12 months of age.

Rabbits can present an anesthetic challenge to researchers and clinicians,^26^, ^27^ with a mortality risk 14 times higher than dogs.^28^ Being a prey species, rabbits are easily stressed which can have significant adverse cardiopulmonary effects. Adequate acclimatization of their surrounds, animal care staff and researchers interacting with the animals should be followed to reduce stress on the animal prior to surgery. Buprenorphine and midazolam premedication combination provides good sedation prior to induction which facilitates stress‐free handling of the rabbit. Appropriate premedication significantly reduces the amount of isoflurane required for general anesthesia. We premedicate at least 45 minutes prior to commencing surgery given the prolonged onset of action of buprenorphine in rabbits; ensuring the rabbits have effective analgesia at the time of surgical intervention. As a benzodiazepine, midazolam provides sedation and muscle relaxation prior to induction. This drug combination reduces the isoflurane requirements for general anesthesia to help reduce the cardiovascular side effects of isoflurane. Additionally, the combined sedative effects of both drugs facilitate stress‐free handling of the rabbit for isoflurane induction. Preoxygenation prior to induction creates an oxygen reservoir that can delay the onset of desaturation which may occur due to isoflurane induction induced apnoea.^29^ Masked isoflurane maintenance has not been associated with adverse events in our experience; however, endotracheal intubation may be of use in establishing and maintaining an airway.

The vasodilative effects of inhalation anesthetics such as isoflurane in combination with their large surface area, make rabbits extremely susceptible to hypothermia.^29^ Hypothermia may result in bradycardia, increased intraoperative hemorrhage, prolonged anesthetic recovery, and postoperative surgical site infection.^29^ We reduce hypothermia as much as possible in our anesthetic protocol by using external heat support (heat mat, towel wrapping, etc) throughout the perioperative period and titrating the isoflurane setting to effect. Additional heat support methods that we could employ include hot water bottles, forced warm‐air blowers, and enclosed humidicribs/incubators.

Rabbits are monogastric herbivores that utilize hindgut fermentation for digestion. Rabbits require a high‐feed intake diet, rich in insoluble fiber to promote gastrointestinal motility and maintain their aradicular hypsodont dentition (high crowned, continuously growing teeth.^30^ High‐quality timothy or oaten hay is ideal and should comprise approximately 80% of the diet. The diet fed to our rabbits aims to meet these requirements and reduce the risk of adverse health, such as the extremely common and potentially fatal condition gastrointestinal stasis (GI stasis). GI stasis is characterized by reduced gastrointestinal motility and the accompanying sequelae of metabolic abnormalities that can result in clinical deterioration of the rabbit. The underlying causes of GI stasis are likely multifactorial and may include low dietary fiber, inadequate water intake, stress, pain, periodontal disease or dehydration secondary to other systemic disease processes. In accordance with our supervising animal ethics committee, we have a comprehensive standard operating protocol for identification, treatment and prevention of GI stasis in our rabbits. Following diagnosis, prompt treatment is initiated to restore appetite, correct electrolyte imbalances, correct dehydration, stimulate gastric emptying, and promote normal gastrointestinal motility. Our treatment protocol is multifactorial, including fluid therapy, prokinetic medications, antibiotics, analgesia, and nutrition.

Our rabbit housing facilities were developed in accordance with the guidelines of the ARRP.^18^ Housing pens are floored with good quality hay, which has been shown to reduce the incidence of pododermatitis and trichobezoars in rabbits.^31^ We seldom see respiratory disease in our rabbit cohort, which may be due to the superior ventilation of pens compared to cages, in conjunction with regular daily cleaning and appropriate screening of symptomatic rabbits.^32^ Laboratory rabbits prefer to be housed with littermates, spending approximately 79% of their time in each other's company.^31^ We maintain 3 to 4 rabbits per pen, each with their own shelter to help minimize conflict. When noted, offending rabbits may be housed singularly with clear vision of other rabbits and/or rotated between pens. In our experience, appropriate stocking densities, environmental enrichment and strictly using female rabbits, reduces conflict between rabbits and encourages the rabbits to display their innate behaviors.

*Pasteurella multocida*, a virulent and readily transmitted gram‐negative coccobacillus, may manifest as multiple clinical syndromes in rabbits including dacrocystitis, rhinitis, pneumonia, otitis media, pyometra, orchitis, abscesses, and septicaemia.^33^, ^34^ Most adult rabbits are believed to be infected with *P. multocida*,^35^ although infection may be asymptomatic.^36^ *P. multocida* has variable and inconsistent antimicrobial sensitivities. Enrofloxacin is a relatively safe, concentration‐dependent fluoroquinolone with activity against *P. multocida* in rabbits^37^; however, multiple studies have questioned its effectiveness.^35^, ^38^ Anecdotally, despite prophylactic Enrofloxacin administration, we have observed occasional *Pasteurella*‐associated abscessation in rabbit PLF studies. For these reasons, we recently updated our perioperative antibiotic choice to Procaine penicillin, which has proven efficacy against *P. multocida*.^39^ Regardless of the antibiotic choice, strict environmental control, good husbandry conditions and adherence to principles of aseptic surgical technique, are in our opinion, the best control measures of *P. multocida*‐associated disease in experimental rabbit cohorts.

## 5 CONCLUSION

We describe an updated protocol on the long‐standing PLF model of spinal fusion from the preoperative to postoperative setting, the variation in the number of lumbar vertebrae in rabbits and 12 week autograft fusion rates. Our techniques accommodate for the unique anesthetic, dietary and husbandry requirements of rabbits, supported by meticulous surgical technique to minimize surgical complications to achieve optimal experimental outcomes. We hope this information is useful for other researchers using this model with the aim of animal welfare preservation and the principles of reduction, refinement and replacement.

## CONFLICT OF INTEREST

The authors declare no potential conflict of interest.

## AUTHOR CONTRIBUTIONS

All authors have read and approved the final submitted manuscript. The following is the author contribution: James D. Crowley, Rema A. Oliver, and William R. Walsh: substantial contributions to research design, data acquisition, analysis, interpretation of current literature, manuscript preparation and critical appraisal; John W. Rawlinson, Rebekah A. Crasto, James M. O'Connor, and Gregory J. Mitchell: animal husbandry procedures, data acquisition; Daniel J. Wills, Michael J. Dan: data acquisition, drafting of manuscript and critical appraisal; Christopher J. Tan: drafting of manuscript and critical appraisal.

## Supporting information


**Appendix S1**: Supporting information.

**Appendix S2**: Supporting information.
