# Supplementary material for: Single level posterolateral lumbar fusion in a New Zealand White rabbit (Oryctolagus cuniculus) model: Surgical anatomy, operative technique, autograft fusion rates, and perioperative care
Source: JOR Spine. 2020 Dec 23;4(1):e1135. doi: 10.1002/jsp2.1135 (PMC7984023; doi:10.1002/jsp2.1135)
Supplement: Supplementary file 1 — Appendix S1: Supporting information. [file JSP2-4-e1135-s002.pdf]

SORL RABBIT PRE-OPERATIVE CHECK & ANAESTHETIC & OPERATIVE RECORD

Stick barcode sticker here

barcode verified ☐ Initials:______

Animal ID:........................**Study ID:**.................................**ACEC approval No.:**.........................**: Sex:**...............**weight (kg):** ....

Heart/lung sounds (circle): normal / abnormal, comment if abnormal:..

Demeanour (circle): normal / abnormal, comment if abnormal: ....

Declaration that animal appears healthy and fit for purpose: Initials: .... **date:**..................

☐ Pre-operative Procaine Penicillin: 50;000 IU/kg *sc/im* (1mL) administered:

Initials:………….. ☐ L

Date: ……………. ☐ R

Procedure:..**Surgery date:**........................**Surgeon 1:**..**Surgeon 2:**..

Premedication: 0.5 ml Temgesic (buprenorphine, 0.03-0.05mg/kg) **+** 0.25ml Hypnovel (midazolam, 0.3-0.5mg/kg) *im*. **time administered:_______ Initials:_______**

**Time**	**Isofluorane (%)**	**Oxygen (L)**	**HR (/min)**	**RR (/min)**	**SpO_2 (%)**	**Eye reflex**	**MM colour**	**CRT**	**Comments**	**Initials** (per time point)

Recovery:

Righting reflex observed (circle): yes/no time: ______

Postural reflexes observed: yes/no time: ______

Ambulation observed: yes/no time: ______

☐ Hartmanns fluids (20mL/kg) (sc) given

Post-operative medication (tick one)

☐ Carprofen (4mg/kg) 0.28 *sc*

☐ Meloxicam (0.85mg/kg) 0.7mL *sc/im*

Initials:…………..

Date: …………….
