# Supplementary material for: Single level posterolateral lumbar fusion in a New Zealand White rabbit (Oryctolagus cuniculus) model: Surgical anatomy, operative technique, autograft fusion rates, and perioperative care
Source: JOR Spine. 2020 Dec 23;4(1):e1135. doi: 10.1002/jsp2.1135 (PMC7984023; doi:10.1002/jsp2.1135)
Supplement: Supplementary file 2 — Appendix S2: Supporting information. [file JSP2-4-e1135-s001.pdf]

# *SORL ACUTE POST-OPERATIVE MONITORING RECORD - rabbit*

**ANIMAL ID:** **Study ID:** **ACEC no:**

**Strain, age, sex:** **Procedure:**

| Emergency Contacts | Name & contact (mobile) number |
|---|---|
| Contact 1: | |
| Contact 2: | |

| | | Day 1 | | Day 2 | | Day 3 | | Day 4 | | Day 5 | | Day 6 | | Day 7 | |
|---|---|---|---|---|---|---|---|---|---|---|---|---|---|---|---|
| | Date: | | | | | | | | | | | | | | |
| | Time: | | | | | | | | | | | | | | |
| | Observer's initials : | | | | | | | | | | | | | | |
| Clinical exam. | Possible abnormal findings (what to look for) | Normal | Abnormal* | Normal | Abnormal* | Normal | Abnormal* | Normal | Abnormal* | Normal | Abnormal* | Normal | Abnormal* | Normal | Abnormal* |
| Posture | Hunched | | | | | | | | | | | | | | |
| Activity (Provoked and unprovoked) | Reduced (especially relative to other post-op) animals) | | | | | | | | | | | | | | |
| Ambulation | Dragging limbs, asymmetry, tremor | | | | | | | | | | | | | | |
| Respiratory pattern | Laboured or rapid | | | | | | | | | | | | | | |
| Fecal/ urinary output | No output | | | | | | | | | | | | | | |
| Body condition | Prominent vertebral spinous processes, scapulae, pelvis | | | | | | | | | | | | | | |
| Surgical wound | Swelling, redness, discharge, disrupted sutures | | | | | | | | | | | | | | |
| Feeding and behaviour | Not feeding, stressed or anxious | | | | | | | | | | | | | | |
| Administer every 48hrs/finishing day 7<br>Procaine Penicillin: 50;000 IU/kg *sc* (1mL) | | | | N/A | | | | N/A | | | | N/A | | N/A | |
| Carprofen (C):<br>Day 1: (4mg/kg) 0.28mL *sc*<br>Day 2 & 3: (2mg/kg) 0.14mL sc | Meloxicam (M):<br>(1mg/kg)<br>0.7mL *sc/im* | C / M<br>Please circle | | C / M<br>Please circle | | C / M<br>Please circle | | N/A | | N/A | | N/A | | N/A | |

***Criteria for veterinary intervention:**
Any signs of abnormal for the clinical signs listed above
Self-trauma and gut stasis (if they manifest)
Signs of distress based on change in local social interaction
Dietary anomalies

**Criteria for euthanasia:**
Paralysis
Respiratory distress
Reduced mobility & not responding to veterinary treatment
Evidence of Fracture
